# Genome Sequences of Plant-Associated *Rhodococcus* sp. Isolates from Tunisia

**DOI:** 10.1128/MRA.00293-20

**Published:** 2020-06-04

**Authors:** Sabrine Dhaouadi, Joe Win, Amira Hamdane Mougou, Adeline Harant, Sophien Kamoun, Ali Rhouma

**Affiliations:** a Laboratory of Bio Aggressors and Integrated Pest Management, Department of Plant Health and Environment, National Institute of Agronomy, University of Carthage, Tunis, Tunisia; b The Sainsbury Laboratory, University of East Anglia, Norwich, United Kingdom; c Partnership for Research and Innovation in the Mediterranean Area (PRIMA), Barcelona, Spain; Broad Institute

## Abstract

The draft genome sequences of plant-associated *Rhodococcus* spp. from Tunisia are reported here. Two Rhodococcus fascians strains were obtained from almond rootstocks, and one Rhodococcus kroppenstedtii strain was obtained from a pistachio tree. The fourth *Rhodococcus* sp. strain was isolated from an ornamental plant.

## ANNOUNCEMENT

Plant-pathogenic *Rhodococcus* spp. are known to cause disease on herbaceous and woody species (1–3). On ornamental plants, major disease symptoms caused by Rhodococcus fascians were described as leafy galls and stem fasciation (4), while on woody trees, the symptoms included stunted growth and proliferation of misshapen shoots (2, 3). Four distinct *Rhodococcus* species isolates from pistachio and almond rootstocks and ornamental plants in Tunisia were used in this study, one of which was shown to cause disease on ornamental plants (5). It is well known that the population structure of *R. fascians* tends to be diverse from one host to another and from one region to another (6). Here, we provide insight into the genetic diversity of plant-associated *Rhodococcus* sp. isolates in Tunisia through their genome sequencing and assemblies.

*Rhodococcus* strains (Table 1) were isolated from plant tissues following the protocol used by Dhaouadi et al. (5) and grown at 27°C on agar plates of D2 medium (7). Genomic DNA extraction and sequencing were outsourced to MicrobesNG (Birmingham, UK). Briefly, three beads were washed with DNA extraction buffer containing lysozyme and RNase A and incubated for 25 min at 37°C. Proteinase K and RNase A were added and incubated for 5 min at 65°C. Genomic DNA was purified using an equal volume of solid-phase reversible immobilization (SPRI) beads (ABM, Richmond, Canada) and resuspended in EB buffer (10 mM Tris-Cl, pH 8.5). DNA was quantified in triplicate with the Quant-IT double-stranded DNA (dsDNA) high-sensitivity (HS) assay in an Eppendorff AF2200 plate reader. Genomic DNA libraries were prepared using a Nextera XT library prep kit (Illumina, San Diego, CA, USA) following the manufacturer’s protocol. DNA quantification and library preparation were carried out on a Hamilton Microlab STAR automated liquid-handling system. Pooled libraries were quantified using the Kapa Biosystems library quantification kit for Illumina on a Roche light cycler 96 quantitative PCR (qPCR) machine. Libraries were sequenced on the Illumina HiSeq instrument using a 250-bp paired-end protocol. The reads were trimmed using Trimmomatic version 0.39 (8) with a sliding window quality cutoff of Q15. Sequence reads were assembled into contigs using SPAdes version 3.7 (9). The assembly metrics in Table 1 were calculated using QUAST version 5.0.2 (10). The genomes were annotated with Prokka version 1.14.3 (https://github.com/tseemann/prokka). Protein-coding features and tRNA were predicted using Prodigal version 2.6 (11), and rRNA was predicted using ARAGORN version 1.2 (12). For taxonomic identification of the bacterial genomes, we used the average nucleotide identity (ANI) test (13). Default settings were used for all software unless otherwise specified.

**TABLE 1:** Summary statistics for *Rhodococcus* genomes assembled from Illumina reads^*a*^

Organism	Host	Host common name	BioSample accession no.	No. of contigs	Largest contig (bp)	Total length (bp)	GC content (%)	Mean coverage (×)	No. of reads	*N*_50_ (bp)	No. of CDS	No. of tRNAs	No. of tmRNAs	GenBank accession no. (assembly)	SRA accession no.
Rhodococcus fascians GS6	Prunus dulcis	Bitter almond	SAMN13734959	122	318,389	5,441,276	64.56	40.6029	552,329	120,644	5,112	51	1	JAAFYX000000000	SRR11109608
Rhodococcus fascians SB10	Prunus persica × Prunus amygdalus	Garnem rootstock	SAMN13734960	199	352,664	5,548,817	64.39	54.8541	703,905	133,430	5,158	50	1	JAAFYW000000000	SRR11109607
*Rhodococcus* sp. strain B10	Iresine herbstii Hook	Herbst’s bloodleaf	SAMN13734961	156	338,193	5,495,644	65.11	109.753	1,580,360	112,702	5,154	51	1	JAAFYV000000000	SRR11109606
Rhodococcus kroppenstedtii K5	Pistacia vera L. cv. Mateur	Pistachio	SAMN13734962	157	168,870	4,040,887	70.26	84.8393	836,282	57,161	3,697	51	1	JAAFYU000000000	SRR11109605

a CDS, coding DNA sequences; tmRNA, transfer-messenger RNA.

The assembly statistics and total number of genes are shown in Table 1. The results based on the ANI test and current taxonomic nomenclature revealed an identity of over 90% of the submitted genome sequence to *Rhodococcus* species. The sequence of the isolate K5 genome is 97.9% identical to the type strain of Rhodococcus kroppenstedtii, the isolate B10 genome is identical to multiple *Rhodococcus* type strains, the isolate SB10 genome is 97.7% identical to Rhodococcus fascians NBRC 12155, and the isolate GS6 genome is 97.8% identical to Rhodococcus fascians NBRC 12155.

### Data availability.

This whole-genome shotgun project has been deposited at DDBJ/ENA/GenBank under accession numbers JAAFYU000000000, JAAFYV000000000, JAAFYW000000000, and JAAFYX000000000. The versions described in this paper are JAAFYU010000000, JAAFYV010000000, JAAFYW010000000, and JAAFYX010000000. The raw sequence reads have been deposited in the NCBI Sequence Read Archive under BioProject number PRJNA598862 and run numbers SRR11109605, SRR11109606, SRR11109607, and SRR11109608.
